# Craniosynostosis in Siblings, an Extremely Rare Occurrence: A Case Report

**DOI:** 10.1002/ccr3.9617

**Published:** 2024-11-23

**Authors:** Tirth Bhavsar, Sachin Mahendrakumar Chaudhary, Sumesh Singh

**Affiliations:** ^1^ Smt. NHL Municipal Medical College Ahmedabad Gujarat India; ^2^ GCS Medical College, Hospital and Research Center Ahmedabad Gujarat India; ^3^ Institute of Medicine Tribhuvan University Kathmandu Nepal

**Keywords:** craniosynostosis, dolichocephaly, midface hypoplasia, neurological development, proptosis, siblings

## Abstract

Craniosynostosis (CS) is the premature fusion of skull sutures, with all sutures except the metopic suture typically fusing in adulthood. Premature fusion constrains brain growth, leading to abnormal skull shape and potential neurocognitive or neurological issues, along with syndromic features in some cases. While CS is rare, its occurrence in siblings is exceptionally uncommon and holds significant academic importance. We report a case of CS in siblings: a 13‐month‐old boy and his five‐and‐a‐half‐year‐old sister. Neither parent exhibits craniofacial dysmorphism or signs of increased intracranial pressure (ICP). The younger sibling presents with dolichocephaly and normal neurological, cognitive, and motor development, while the elder sibling exhibits proptosis, midface hypoplasia, and normal developmental milestones. Neither sibling displays limb or systemic anomalies. Imaging studies, including multislice plain CT brain with 3D skull reconstruction and MRI, revealed multiple suture closures. The younger sibling has complete sagittal suture closure with partial closure of other sutures, while the elder sibling shows multisutural CS. Ophthalmologic evaluations and developmental assessments excluded increased ICP and systemic issues. Most CS cases follow an autosomal dominant inheritance pattern, making this case particularly significant. CT with 3D skull reconstruction remains the diagnostic gold standard. Management aims to preserve cosmetic appearance and prevent complications from increased ICP. Treatment options range from conservative follow‐up to surgical interventions, including endoscopic suturectomy, open craniotomy, and distraction osteogenesis, depending on the presence of neurocognitive issues or elevated ICP. Both siblings currently show normal neurological, cognitive, and motor development without increased ICP, emphasizing the need for ongoing monitoring to identify new developments or recurrence after treatment. Differential diagnoses, such as deformational plagiocephaly, must also be considered in such cases.

## Graphical abstract

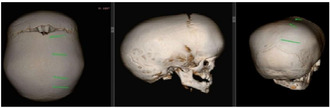

**Summary**

- This report highlights an exceptionally rare case of craniosynostosis occurring in siblings, with phenotypically normal parents.
- It underscores the importance of comprehensive genetic evaluation and ongoing monitoring to prevent complications and support normal neurological and cognitive development in affected individuals.

## 1 Introduction

Craniosynostosis (CS) is the early fusion of skull sutures during development, resulting in esthetic deformities of the skull and face. This condition can also lead to restricted brain growth at a young age and increased pressure within the skull, potentially affecting the central nervous system. Furthermore, patients with syndromic CS have dysmorphic traits that are characteristic of the related disorders.

The fusion of the sagittal and lambdoid sutures typically does not commence until the individual reaches the age of 18 [1]. The coronal suture often initiates fusing at its lower end during the early years of the second decade of life, although it does not fully finish the fusion process until the age of 18. The metopic suture often initiates fusion around 3 months of life, while complete fusion may not occur until after 2 years of age [1].

The prevalence of CS is approximately 1 in 2000 live births. The likelihood of a sibling being born with the same condition as a child with CS is between 0% and 4% [2]. Children of parents with CS are also at a comparable risk. The sagittal CS is the predominant form, accounting for 50%–58% of cases of nonsyndromic CS [3]. It has a prevalence rate of roughly 1 in 5000 newborns [4, 5]. Coronal synostosis is the second most prevalent kind of nonsyndromic CS, with an occurrence rate of roughly 1 in 10,000 births [4, 6]. The prevalence of metopic synostosis is estimated to be around 1 in 15,000 live births [7]. Lambdoid synostosis has a prevalence of roughly 1 in 33,000 births [8], making it quite rare. The prevalence is typically 2%–4% [9].

We are describing a case of CS among siblings, as this is an exceptionally uncommon event when it occurs in siblings. The siblings as well as the parents did not exhibit characteristics of syndromic CS. This pedigree analysis does not support autosomal dominant inheritance. All genetic variations of CS, with the exception of Carpenter syndrome and Antely‐Bixler syndrome, GAPO (growth retardation, alopecia, pseudoanodontia, and optic atrophy) syndrome, and Crouzon‐like CS disorder, exhibit an autosomal dominant inheritance pattern [10]. Both the siblings having CS, may support the hypothesis of autosomal recessive inheritance. Prenatal genetic diagnosis is possible if there has been a previously afflicted sibling or parent. There are currently approximately 70 reported cases of Carpenter syndrome and around 30 reported cases of Antley‐Bixler syndrome documented in the global literature [11]. Both of these conditions are autosomal recessive. These syndromes are characterized by specific symptoms, and both the siblings did not exhibit any of these features. These facts render this case report of utmost academic significance.

## 2 Case History/Examination

A case of CS in siblings: a 13‐month‐old brother and a five‐and‐a‐half‐year‐old sister was presented in our clinic. Neither parent exhibited any craniofacial dysmorphic features, neurocognitive or intellectual deficiencies, symptoms or signs of increased intracranial pressure (ICP) associated with CS, or any syndromic CS features. It is noteworthy that the maternal history reveals no exposure to teratogens during either pregnancy, including valproate and fluconazole. The mother did not take any medications aside from standard prenatal supplements, specifically iron, calcium, folic acid, and vitamin B12. The parents had not undergone genetic testing for potential mutations associated with CS. This decision was influenced by their lower middle‐class economic status and the absence of any phenotypic characteristics suggestive of the condition in either parent. There was no family history for CS on maternal or paternal side.

Both pregnancies progressed without complications, and the deliveries occurred at full term. The infants did not require additional postnatal care or treatment for complications. Birth weights were within normal ranges: the younger male child weighed 3.2 kg, while the elder female child weighed 2.8 kg. Maternal age at the time of the second delivery was 32 years, while paternal age was 36 years.

**FIGURE 1:**
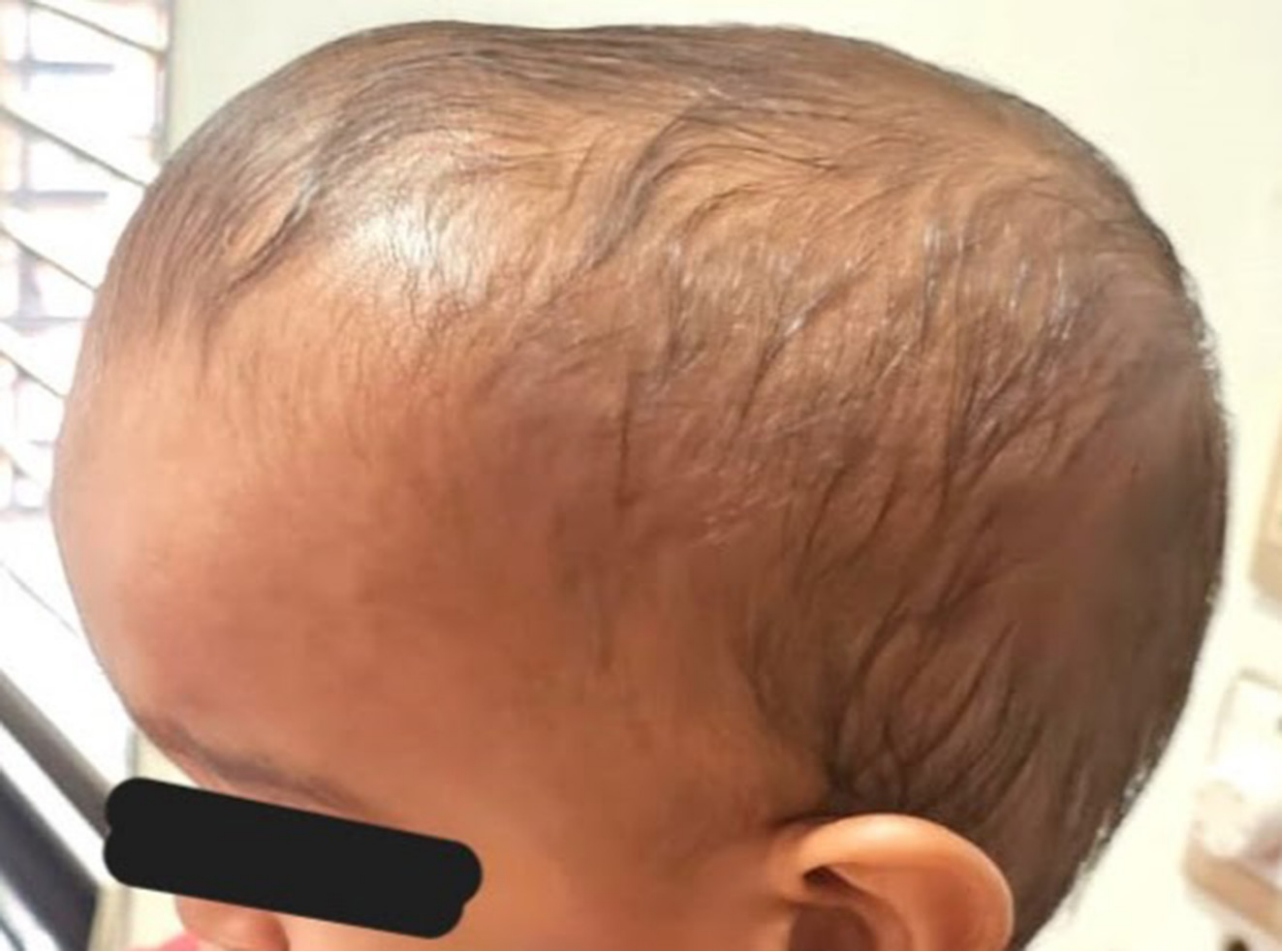
Image of a 13‐month‐old child exhibiting characteristic features of craniosynostosis, including dolichocephaly with a wide and high forehead, small nose with a low bridge, and midface hypoplasia. The child demonstrates normal neurological function, cognitive and motor development, with no signs of increased intracranial pressure or systemic anomalies.

**FIGURE 2:**
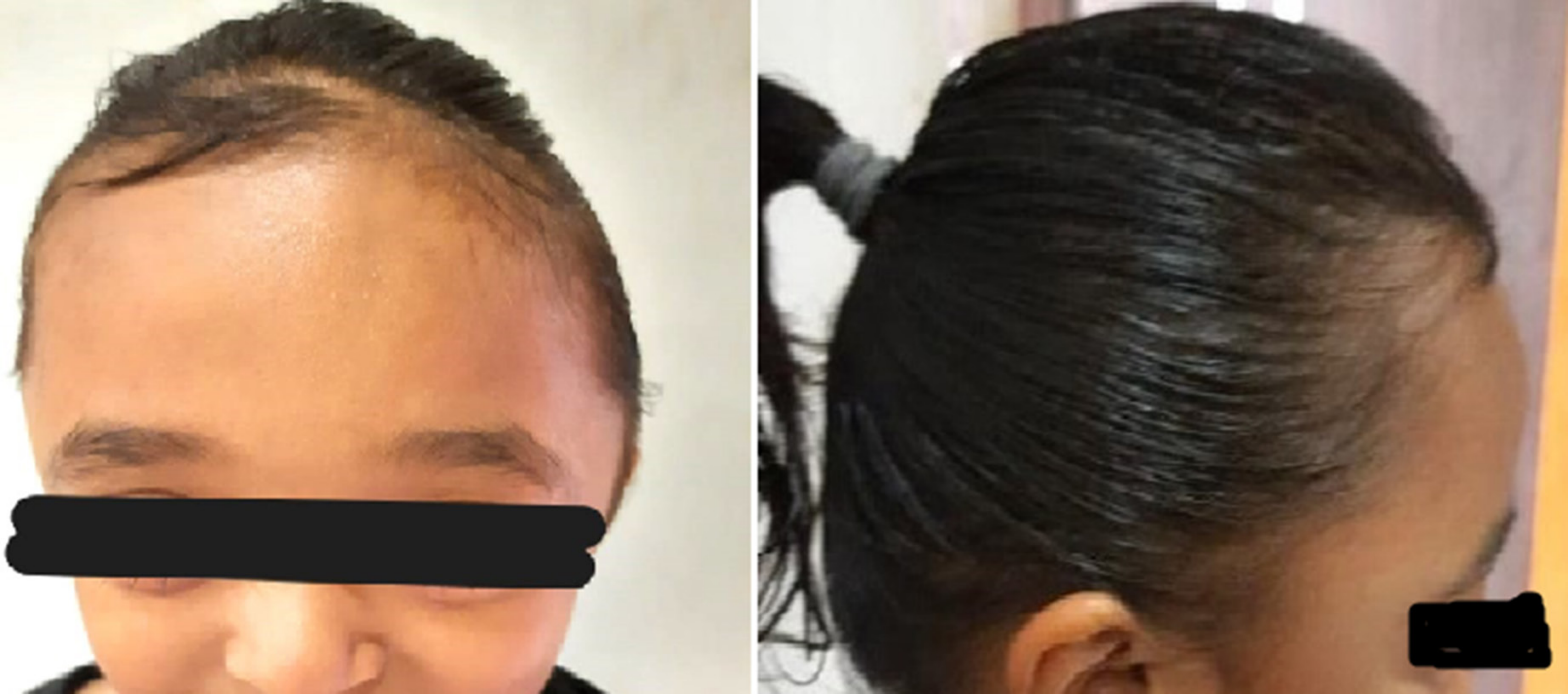
Image of a 5.5‐year‐old sibling exhibiting features of craniosynostosis, including proptosis, a wide and high forehead, low nasal bridge, and midface hypoplasia. The child shows normal neurological function, cognitive and motor development similar to her brother.

The younger brother presented with dolichocephaly, characterized by a wide and high forehead, a small nose with a low bridge, and midface hypoplasia (Figure 1). All other features were normal, including normal neurological function, cognitive and motor development, absence of increased ICP, and no anomalies in limbs, fingers, toes, or any systemic issues (Table 1). The elder sister exhibited proptosis, a wide and high forehead, a low nasal bridge, and midface hypoplasia (Figure 2). Similar to her brother, she had normal neurological function, cognitive and motor development, no signs of increased ICP, and no limb, finger, toe, or systemic anomalies (Table 1).

**TABLE 1:** Characteristic features of craniocynostosis (CS) and MRI findings in the siblings.

Parameter	Male child	Female child
Age	13 months	5 years and 6 months
Fused sutures	Complete closure and obliteration of midline sagittal suture. Early partial obliteration of bilateral lambdoid, occipito‐mastoid and squamosal sutures. Age expected complete fusion of metopic suture. Coronal suture not closed. Anterior fontanelle prominent	Sagittal suture fused. Bilateral coronal, lambdoid, spheno‐frontal, squamous and occipitomastoid suture fused. Spheno‐squamous suture partially fused. Multisutural craniosynostosis
Presenting symptoms	No symptoms other than cosmetic disfigurement of head, no symptoms related to musculoskeletal, cardiac, pulmonary system	No symptoms
Cranio‐facia features	Dolicocephaly—(Figure 1)	(Figure 2)
Neurocognitive development	At present 18 months of age language mile stone are normal—able to speak single words (holophrastic stage of speech) Motor mile stone—Capable of ambulating effortlessly while grasping the finger of another person using only one hand Able to feed with fingers	All mile stones are achieved as per age. Early school performance is at par and good.
Symptoms of increased intracranial pressure (ICP)	Nil	Nil
Signs of increase ICP	Anterior fontanelle open. No papilledema	No papilledema
Syndromic features of CS	Nil—Hands, feet, fingers and toes are normal	Nil—Hands, feet, fingers and toes are normal
Head circumference	Within normal range for age as per WHO chart	Within normal range as per WHO chart
MRI	No chiari‐I malformation or ventriculomegaly	Altered contour of cerebral hemisphere on left side. Optic nerve tortuous with prominence of perioptic cerebrospinal fluid (CSF) and posterior orbital sclera flattening bilaterally with empty sella favoring changes of increased ICP No chiari‐I malformation or ventriculomegaly
Treatment	Not given	Not given

## 3 Methods

### 3.1 Differential Diagnosis

Differential diagnosis for CS includes deformational plagiocephaly, positional molding, and other craniofacial anomalies. It is crucial to differentiate CS from these conditions as they present with overlapping features. Deformational plagiocephaly, often due to external pressure on the skull, does not involve suture fusion and can be managed conservatively. A thorough clinical examination and imaging studies, such as CT or MRI, help distinguish CS from other causes of cranial deformities.

### 3.2 Investigation

**FIGURE 3:**
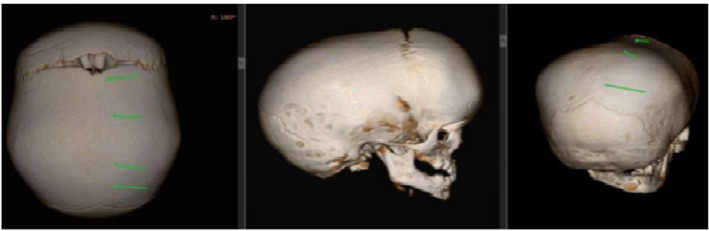
CT 3D reconstruction of skull of the male sibling.

**FIGURE 4:**
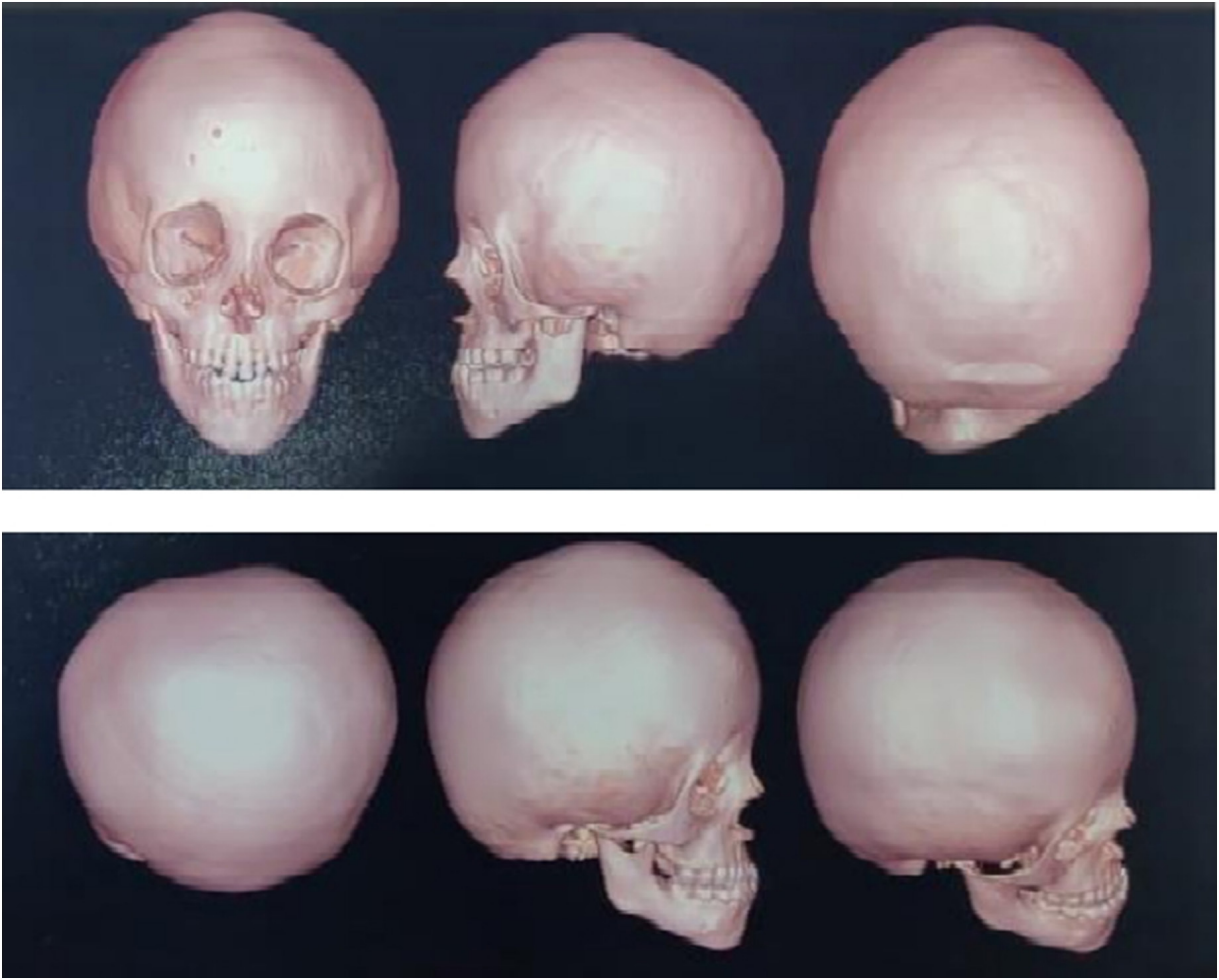
CT 3D reconstruction of skull of the female sibling.

Both siblings underwent comprehensive imaging studies, including multislice plain CT brain with 3D CT skull (CS protocol) and MRI brain, to confirm the diagnosis and evaluate the extent of suture fusion (Figures 3 and 4). The CT scans revealed complete closure and partial obliteration of multiple sutures in both children, detailed in Table 1. Routine blood tests, developmental assessments, and ophthalmologic examinations were conducted to rule out increased intracranial pressure and other systemic anomalies.

### 3.3 Treatment

Given the absence of symptoms related to increased ICP and normal developmental milestones, both siblings were managed conservatively with regular monitoring. Surgical intervention was not deemed necessary at this stage. The focus was on maintaining normal neurocognitive development and preventing potential complications. Future treatment plans include periodic evaluations and possible surgical considerations if symptoms of increased ICP or developmental delays emerge. Genetic counseling and evaluation were advised for the family to better understand the potential hereditary aspects of the condition.

## 4 Conclusion and Results

CS is a rare condition, with CS in siblings being extremely rare. Genetic evaluation and counseling should be offered to affected families and parents. Prenatal diagnosis is possible. CT with 3D skull reconstruction remains the gold standard for diagnosis. Treatment aims to maintain cosmetic appearance, preserve neurocognitive and language development, and prevent complications from increased ICP.

In this case, neither parent displays phenotypic features of CS, yet both siblings exhibit phenotypic features without limb, finger, toe, or systemic anomalies, making this case of significant academic interest.

## 5 Discussion

CS manifests in various forms. Primary CS arises from premature sutural ossification. Secondary CS is due to underlying abnormalities in brain growth from various etiologies, such as hypothyroidism, rickets, mucopolysaccharidosis, sickle cell anemia, or shunted hydrocephalus. In these cases, the brain's lack of growth prevents increased ICP from driving skull bone enlargement. A third type is syndromic CS, with over 180 identified syndromes, including Apert syndrome (syndactyly), Crouzon syndrome, GAPO syndrome, Carpenter syndrome, and Pfeiffer syndrome (big toe and broad thumb) [12]. These syndromes often involve other systems such as musculoskeletal, cardiac, pulmonary, or genitourinary.

Children with syndromic CS typically have a genetic basis, often resulting from gain‐of‐function mutations in fibroblast growth factor receptor (FGFR) genes, leading to premature sutural fusion. The majority of CS are autosomal dominant with approximately half of these cases being de novo mutations [9]. CS can also be classified as simple when only one suture is affected or compound/complex when multiple sutures are prematurely fused.

In newborns, skull bones are membranous and separated by sutures, allowing passage through the birth canal and accommodating brain growth. Premature suture closure redirects growth perpendicular to the patent sutures, resulting in an abnormally shaped skull and potentially increased ICP, alongside sensory, respiratory, and neurological dysfunctions [4, 13, 14, 15, 16].

Patients with CS may present with skull and facial disfigurement, microcephaly, facial asymmetry, midfacial hypoplasia, hypertelorism or hypotelorism, low‐set or displaced ears or orbits, failure to thrive, signs of increased ICP, ventriculomegaly, neurological deficiencies, gaze palsy, and vision problems. Those with single suture CS often exhibit lower neurodevelopmental scores, language and cognitive problems, academic difficulties, behavioral issues, and learning disabilities [17, 18]. Syndromic CS often involves additional anomalies such as syndactyly or broad digits and systemic abnormalities affecting musculoskeletal, cardiac, pulmonary, or genitourinary systems.

Diagnosis of CS is confirmed by CT head with 3D skull reconstruction, the gold standard. Genetic analysis (FGFR1, FGFR2, FGFR3, TWIST, and FBEN1) is essential to identify genetic causes. Most genetic CS cases are autosomal dominant, with Antley‐Bixler syndrome (characterized by disordered sex development and radio‐humeral synostosis), Carpenter syndrome (polysyndactyly), GAPO syndrome and Crouzon‐like disorder being autosomal recessive [19, 20]. MRI and fundus examinations for papilledema are also necessary, along with additional investigations for systemic involvement in syndromic CS. Prenatal diagnosis via ultrasound around 20 weeks' gestation is possible, though even syndromic cases may not be detected during pregnancy [21]. Differential diagnoses, such as deformational plagiocephaly due to specific positioning, torticollis, muscle hypotonia, or cervical spine abnormalities, must not be ruled out.

Treatment includes conservative approaches and surgery, necessitating a multidisciplinary team. Conservative management with regular follow‐up is sufficient in cases without neurocognitive defects or increased ICP, particularly when few sutures are involved. Surgery is required for multi‐suture CS with increased ICP or neurocognitive defects. Endoscopic suturectomy is preferred for infants under 6 months, often combined with postoperative use of a remodeling helmet. Open craniotomy is favored for infants over 6 months due to more rigid bones, allowing better skull remodeling and reducing helmet use postoperatively [22, 23]. Cranial distracters (distraction osteogenesis) may be employed for slow, steady suture separation in multi‐suture cases. The optimal timing for surgical intervention remains controversial, but neurocognitive and morphological outcomes generally improve post‐surgery [23, 24]. Regular follow‐ups are crucial to monitor for possible suture refusion. In syndromic CS, treatment plans should be adapted based on specific defects.

## Author Contributions

**Tirth Bhavsar:** conceptualization, data curation, formal analysis, project administration, supervision, writing – original draft. **Sachin Mahendrakumar Chaudhary:** data curation, investigation, methodology, resources, writing – original draft. **Sumesh Singh:** data curation, methodology, writing – original draft, writing – review and editing.

## Consent

Written informed consent was obtained from the patients' kin to publish this report.

## Conflicts of Interest

The authors declare no conflicts of interest.

## Data Availability

The data that support the findings of this study are available from the corresponding author upon reasonable request.
